# Neuroticism Mediates the Association between Autistic Traits and Choice Reaction Time among Young Adults

**DOI:** 10.3390/bs14100903

**Published:** 2024-10-07

**Authors:** Kassandra De Jesus Cintron, Xiao Yang

**Affiliations:** Department of Psychology, Old Dominion University, 346G Mills Godwin Life Sciences Building, Norfolk, VA 23529, USA

**Keywords:** autistic symptoms, neuroticism, choice reaction time, ex-Gaussian modeling

## Abstract

Autism spectrum disorder (ASD) is a neurodevelopmental disorder that influences an individual’s cognitive functions and social interaction. While most studies have focused on children and adolescents diagnosed with ASD, elevated levels of autistic traits in subclinical populations may also influence individuals’ daily functioning. Autistic traits are also linked to the Big Five personality. In particular, neuroticism (emotion instability) has been shown to be positively associated with autistic traits, which may contribute to behavioral symptoms of autistic traits. The present study aimed to investigate the association between autistic symptoms and sensorimotor processing among a subclinical population. One hundred young adults (*M_age_* = 20.32 years; *SD* = 3.69 years; 69 female) completed a choice reaction time (RT) task, and their behavioral performance was analyzed using the ex-Gaussian modeling. The Autism Quotient (AQ) and the Ten-Item Personality Inventory (TIPI) were used to assess autistic traits and neuroticism, respectively. The mediation analysis was conducted to examine the behavioral mechanism through which autistic traits influence sensorimotor processing. The results showed that the AQ score was negatively correlated with RT and positively correlated with neuroticism score. Importantly, the mediation analysis indicated an indirect effect, suggesting that neuroticism mediates the association between the AQ score and RT. The findings indicated a possible mechanism of the association between autistic traits and sensorimotor responses and suggested that neuroticism should be included as an intervention target for ASD. The present study contributes to the research on autistic traits and has practical implications for future intervention programs to improve daily functioning among individuals with ASD.

## 1. Introduction

Autism spectrum disorder (ASD) is a neurodevelopmental disorder that is characterized by impairments in communication and other forms of social interaction and repetitive behaviors. ASD affects approximately 1% of children worldwide, and its prevalence in high-income countries is even higher (e.g., 1.5% in Europe and 3% in the U.S.) [1,2]. The global economic burden of ASD was projected to be USD 461 billion in 2025 and will continue increasing in the future [3]. Therefore, ASD poses a major public health challenge.

The American Psychiatric Association (APA) included diagnostic criteria for ASD in the Diagnostic and Statistical Manual of Mental Disorders, the fifth edition (DSM-5) and established that the first category under ASD consists of persistent deficits in social communication and interactions in areas such as social–emotional reciprocity and nonverbal communication [4]. Also, other deficits implicated in ASD include difficulties in developing, maintaining, and understanding social relationships. Furthermore, ASD symptoms involve restricted and repetitive patterns of behavior such as repetitive habits, abnormally intense fixations on interests, and hyperreactivity to sensory stimuli. Those symptoms occur during the early stages of development and result in long-lasting impairments that are clinically significant in social and occupational settings [4].

Despite the well-defined diagnostic criteria for ASD [4], it is often underreported in the population due to a wide range of factors. For example, parents or caregivers may not recognize early signs of ASD and may falsely attribute autistic symptoms to other developmental or behavioral issues [5]. Among adults, social expectations and subjective recall biases may prevent an individual from adequately reporting ASD symptoms [6]. Additionally, cultural differences in social communication also influence the diagnosis of ASD [7]. Therefore, it is important to identify universal behavioral symptoms and traits that are implicated in ASD across different cultures. Interestingly, these undiagnosed behavioral symptoms of ASD may be considered trait-level individual differences in cognition and social behavior.

Autistic traits can be found in the general population, though they are behaviors and personality characteristics that are commonly associated with ASD [8]. Autistic traits influence social communication, sensory perception, emotional regulation, and cognitive processes. It is important to recognize that a participant that exhibits a high level of autistic traits may not necessarily meet the diagnostic criteria of ASD [4]. Autistic traits are not unique to autism and are often found in non-autistic populations with other conditions such as anxiety or schizophrenia [9]. An example of a diagnostic overlap is attention-deficit/hyperactivity disorder (ADHD), as children with the disorder similarly have social impairments [10], communication difficulties [11], behavioral difficulties [12,13], as well as attentional and overactivity problems [14]. Studying autistic traits in subclinical populations will contribute towards minimizing stigma towards autism, as studies often examine whether a deficit extends to those with subclinical autistic traits [9].

The Big Five personality is one of the most influential frameworks of individual differences in behavior [15]. The Big Five Inventory measures an individual’s personality type through their scores in five categories: extraversion, neuroticism, agreeableness, conscientiousness, and openness to experience [15]. A recent study that investigated the association of the Big Five and emotional intelligence with camouflaging behaviors found that neuroticism was positively associated with autistic traits [16]. A meta-analysis showed an association with lower openness, extraversion, agreeableness, conscientiousness, and emotional stability; however, neuroticism was referred to as emotional stability to allow for a consistent direction of effects across the Big Five traits [17]. Moreover, individuals diagnosed with ASD in adulthood had the tendency to display characteristics related to emotional instability, social detachment, and introversion [18]. When using a subclinical population sample, there is support for a significantly positive correlation between overall autistic traits and neuroticism [19,20].

The symptoms of ASD also reflect altered neurobehavioral processes. Excitatory–inhibitory imbalance (E-I imbalance) is a sensorimotor process that has been linked to ASD [21]. E-I imbalances may be influenced by an increase in excitatory neurotransmitters (e.g., glutamate), which promote neural activity, or a decrease in inhibitory neurotransmitters (e.g., GABA), which suppress neural activity [21]. Those changes in the nervous system lead to difficulties in prepotent response inhibition among individuals with ASD [22]. Moreover, in comparison with the healthy population, patients with ASD showed a more rapid deterioration of their inhibitory control abilities as stop-signal delays (SSDs) were increased, suggesting that these individuals have a limited capacity when withholding behavioral responses that increase in severity during longer periods of uncertainty [23]. Altogether, ASD is associated with reduced top-down modulation of sensorimotor systems [24].

One way to assess sensorimotor processing and top-down regulation is to measure reaction times (RTs) in various cognitive tasks. RTs reflect the net effect of information processing at different levels, and speed of processing underlies a wide range of cognitive abilities [25]. Several studies showed that RTs in individuals with ASD were slower than those among healthy controls [24]. Slower RTs among individuals with ASD have been explained by abnormal brain development, and autistic adolescents and adults have shown smaller frontal lobe and decreased white matter density [24]. High levels of autistic symptoms were also positively related to variability in RTs [26]. However, the relationship of ASD or autistic symptoms with RT is not straightforward. Noterdaeme et al. reported a null finding of the association between ASD and response speed [27]. Moreover, during tasks that did not require complex social processing, RTs did not differ between individuals with ASD and neurotypical controls [28].

The mixed findings of the ASD-RT association may be attributed to the metrics of response speed. Specifically, the shape of RT distributions is usually skewed, and thus the traditional measurements, including median and mean RT, are misleading and fail to analyze the variability of RTs at the trial-by-trial level [29,30,31]. To overcome this limitation, several studies have used the ex-Gaussian model to examine RT data [32,33]. The ex-Gaussian model is used to fit the natural distribution of the RT data and separate very slow responses (the exponential tail of the RT distribution) from faster trials in the Gaussian (normally distributed) portion of the data. The components of an ex-Gaussian distribution indicate different aspects of sensorimotor processing. While ex-Gaussian parameters, mu and sigma (μ and σ represent the mean and the SD of the Gaussian component of the RT distribution, respectively), indicate sensory–perceptual processing, the proportion of very slow RTs of the tail estimated with the parameter tau (τ) indexes top-down regulation [30,34]. Therefore, it is possible that behavioral symptoms of ASD are differentially related to separate ex-Gaussian parameters of RT.

### Current Study

The present study aimed to investigate the association between autistic symptoms and sensorimotor processing using the ex-Gaussian approach. While most studies have focused on children diagnosed with ASD, there is little research that specifically studies adult populations. Moreover, it is important to establish RT metrics as a tool for ASD diagnosis. Therefore, the present study focused on autistic symptoms among healthy young adults. Importantly, neuroticism, a Big Five trait that reflects the E-I imbalance, was tested as a potential behavioral mechanism mediating the relationship between autistic symptoms and response speed. Autistic symptoms were assessed by the Autism Quotient (AQ) [8], and neuroticism was measured by the Ten-Item Personality Inventory, a short version of the Big Five personality scale [35]. In light of the literature, the following hypotheses were established: AQ scores would be positively correlated with neuroticism scores and positively correlated with the ex-Gaussian parameters σ and τ, and they would be negatively correlated with μ (Hypothesis 1). Furthermore, neuroticism scores would mediate the association of AQ scores with the ex-Gaussian parameters of RT (Hypothesis 2).

## 2. Method

### 2.1. Participants

The sample of this study consisted of 100 participants (*M_age_* = 20.32 years; *SD* = 3.69 years; 69 female) who were recruited from undergraduate psychology courses at Old Dominion University (ODU). The exclusion criteria included histories of neurologic illness and currently taking psychotropic medication. Participants received course credits for their participation. This study was approved by the ODU Institutional Review Board.

### 2.2. Materials and Tasks

The present study is a part of a larger project investigating cognitive mechanisms of psychopathology, including a series of self-report scales. This report focuses on the assessments of ASD symptoms and related trait measures. The adult version of the Autism Quotient (AQ) was used to assess ASD symptoms in the sample of the present study. The AQ is a 50-item self-report questionnaire to measure the level of an individual’s autistic traits, which include five domains: Social Skills, Attention Switch, Attention to Details, Communication, and Imagination [8]. Each item is rated on a four-point scale, and higher AQ scores indicate increasing levels of autistic traits. As a screening instrument, Cronbach’s α of the AQ ranges from 0.70 to 0.90, indicating good internal consistency [8,19,36]. Moreover, the AQ shows moderate to high test–retest reliability and good validity [19,36]. In the current study, Cronbach’s α of the AQ was 0.71, indicating an acceptable internal reliability.

The participants also complete the Ten-Item Personality Inventory (TIPI) [35], which measures the Big Five traits: openness, conscientiousness, extraversion, agreeableness, and neuroticism. Each item in the TIPI is rated on a seven-point Likert scale. The TIPI shows acceptable internal consistency (Cronbach’s α ranges between 0.50 and 0.70) and validity [35,37]. In the current study, Cronbach’s α of the AQ was 0.70, indicating an acceptable internal reliability. Given the focus on the association of ASD with emotional stability, the TIPI neuroticism score was analyzed in the present study. Higher scores on the neuroticism subscale indicate lower levels of emotional stability.

A choice reaction time (RT) task was used to assess sensorimotor processing. In the task, participants were asked to press the left or right arrow key on a computer keyboard as quickly and accurately as possible in response to the task stimuli (left and right arrows) presented on the screen, respectively. Each stimulus was presented for 2.5 s or until we detected key pressing. There were a total of 100 trials, and the order of the trials was randomly counterbalanced. The inter-trial interval was 3 s.

### 2.3. Procedure

Upon arrival at the laboratory, informed consent was obtained from all participants. Participants then completed the questionnaires. Afterwards, participants were comfortably seated in the laboratory, and the choice RT task was introduced to the participants. After ten practice trials, participants completed 100 trials of the choice RT task. At the end of the task, participants were asked to sit still and remain quiet for two minutes as the recovery period, after which participants were thanked and informed about the purpose of this study.

### 2.4. Data Reduction

The behavioral data included response accuracy and reaction time (RT). Response accuracy was computed as the percentage of correct responses in all trials. RT data were analyzed using the ex-Gaussian approach. Specifically, the correct RT trials that were longer than 100 ms in each block included the ex-Gaussian modeling [30,31]. Note that slow RT trials were not excluded, which was different from the traditional calculation of RT metrics. The short RT trials (<100 ms) were deleted to avoid the confounding effects of guessing on sensorimotor responses, which constituted <0.1% of all data for a participant. This approach is consistent with the guideline [30,31,32,33]. The RT distribution of each condition was fitted with the ex-Gaussian model using the “retimes” package in *R* statistical software [38]. To ensure the robustness of the models, bootstrapping with 1000 iterations was used to estimate the ex-Gaussian parameters μ, σ, and τ [30,31]. In addition, mean RTs were also calculated to validate the ex-Gaussian approach.

### 2.5. Analytic Approach

To investigate whether elevated ASD symptoms lead to a fast response speed and fewer attentional lapses, the effects of the AQ score on ex-Gaussian parameters, μ and τ, were estimated in linear regression models. The two ex-Gaussian parameters were entered into separate regression models as the dependent variable. In each model, the AQ was entered as the independent variable, which was controlled for participants’ gender and age. Effect sizes of independent variables were estimated by the partial *R*^2^. Below are the regression equations (terms for intercepts and covariates are not shown for simplicity):

*Ex-Gaussian Parameter* = *c* × *AQ* (1)

*Ex-Gaussian Parameter* = *b* × *Neuroticism* + *c’* × *AQ* (2)

*Neuroticism* = *a* × *AQ* (3)

In the equations, *Ex-Gaussian Parameter* represents μ and τ in sperate models; *AQ* represents the Autism Quotient score; and *Neuroticism* represents the neuroticism score, which served as the mediator. Equation (1) represents the total effects (*c*) of the AQ score on the dependent variable; Equation (2) represents the direct effects (*c’*) of the AQ score on the ex-Gaussian parameter, after accounting for the effect (*b*) of neuroticism; and Equation (3) represents the effects (*a*) of the AQ score on neuroticism.

Mediation analysis of indirect effects was used to examine whether neuroticism is a mediator through which ASD symptoms influence response speed and attentional failures [39,40]. The indirect effects were calculated from the parameters in Equations (2) and (3) as *a* × *b*. *Monte Carlo* simulation was then used to determine the 95% confidence interval of each indirect effect [40,41].

## 3. Results

The demographic and descriptive statistics are displayed in Table 1. Compared to male participants, female participants showed higher neuroticism scores and ex-Gaussian sigma (see Table 1).

Relationships among variables are shown in Table 2. The AQ was positively correlated with neuroticism scores and negatively correlated with the ex-Gaussian parameter μ. Moreover, response accuracy was positively correlated with μ and negatively correlated with τ, and the traditional mean reaction time was positively correlated with all ex-Gaussian parameters (see Table 2).

**Figure 1:**
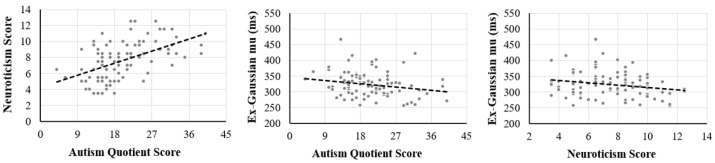
Scatterplots of the relationships among Autism Quotient score, neuroticism score, and ex-Gaussian mu. The dashed lines in the figure represent the unadjusted lines of best fit.

The relationships among variables in the regression models are shown in Figure 1. The regression model of ex-Gaussian parameters accounted for a significant portion of variance of the dependent variable, *F*(3, 96) = 1.23, *p* = 0.040. The results showed that a higher AQ score was correlated with lower ex-Gaussian parameters μ, β = −1.223, *SE* = 0.531, *p* = 0.024, partial $R_{\beta }^{2}$ = 0.043, but was not related to τ, β = −0.408, *SE* = 0.378, *p* = 0.283. The results indicated an association between the ASD symptom level and sensorimotor response speed. However, the regression models of AQ scores and other ex-Gaussian parameters (σ and τ) were not significant.

**Figure 2:**
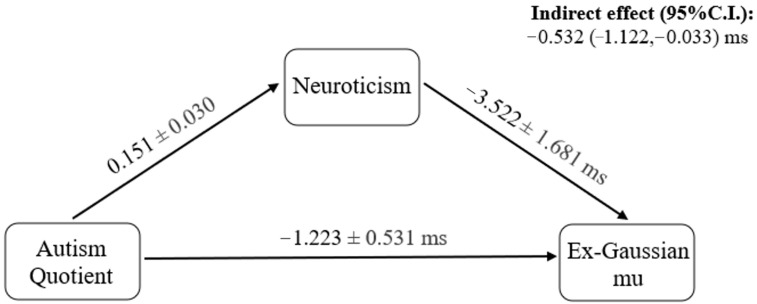
Mediation of neuroticism, through which the Autism Quotient was negatively associated with the ex-Gaussian parameter mu. Regression coefficients and standard errors were presented in the model.

In the mediation analysis, the AQ score was positively associated with the TIPI neuroticism score, β = 0.151, *SE* = 0.030, *p* < 0.001, partial $R_{\beta }^{2}$ = 0.222. Moreover, the neuroticism score predicted μ, β = −3.522, *SE* = 1.681, *p* = 0.039, partial $R_{\beta }^{2}$ = 0.023, but not τ, β = 0.378, *SE* = 1.197, *p* = 0.753. Based on the linear regression models, there was an indirect effect of the AQ score on ex-Gaussian parameters μ through the TIPI neuroticism score, *a* = 0.151, *b* = −3.522, 95% *C.I.* = (−1.122, −0.033), indicating a mediation of neuroticism through which ASD symptoms speed up sensorimotor responses (see Figure 2).

## 4. Discussion

The present study examined the effects of autistic symptoms on sensorimotor processing, and the potential mechanism through which ASD influences motor responses. A choice RT task was used to evaluate sensorimotor processing. Moreover, ASD symptoms and neuroticism were assessed by the AQ and TIPI, respectively. The ex-Gaussian approach was used to derive the parameters that differentially indicate lower-level perceptual processing, response variability, and controlled attentional processes. Consistent with the hypotheses, the AQ score was negatively correlated with RT and positively correlated with the neuroticism score. Importantly, the mediation analysis indicated an indirect effect, suggesting that neuroticism mediates the association between the AQ score and RT.

The first hypothesis was supported. The present results indicate that, among healthy college students, higher levels of ASD symptoms predicted a faster response speed. Prior studies of ASD reported mixed findings of the response speed and ASD [24,25,26,27,28]. A meta-analysis summarized studies using various experimental tasks and found that, while ASD increases RT variability, there were no significant overall differences between ASD individuals and healthy controls [42]. The inconsistencies in the literature of ASD and RT might be due to the diverse RT tasks and the assessments of RT responses in those studies. As opposed to complex cognitive tasks [24], we used the choice RT task, which was less cognitively demanding. Moreover, our RT data were analyzed using ex-Gaussian modeling, which helps clarify the discrepancy in the meta-analysis, as slow RT trials may mask the effect of ASD on the parameter mu. The effects of ASD on mu, but not tau, may also contribute to elevated RT variability among individuals with ASD [26]. Moreover, it is worth noting that the sample in the present study was made up of healthy individuals, and the AQ score indicated preclinical autistic symptoms. When considering elevated autistic traits among individuals with ADHD and other forms of behavior disorders [10,11,12,13,14], the present findings of the association between AQ scores and ex-Gaussian mu may reflect altered behavioral mechanisms that are linked to attentional processes but may not mirror the genuine effects of ASD as a mental disorder.

The present results of neuroticism and RT further support the notion that different behavioral components of ASD may differentially contribute to impaired attentional processes. Neuroticism, or emotion instability, has been linked to both faster and slower response speeds [43]. The specific effect of neuroticism on RT is determined by the characteristics of the RT task. In the present study, the choice RT task did not require complex processing of the RT stimuli, and a shortened RT related to a high neuroticism score reflected increased impulsivity and reduced inhibition control. Moreover, high levels of neuroticism have been explained by elevated levels of excitatory neurotransmitters in the central nervous system, which echoes the hypothesis of E-I imbalance [21]. Specifically, not only does E-I imbalance underlie ASD but it also reduces inhibition control and contributes to neuroticism. In that regard, neuroticism and ASD seem closely related to each other, which is in line with the previous literature [18].

Importantly, the mediation model (H2) was supported by the results, suggesting that instable emotionality among individuals with more ASD symptoms leads to a faster response. A shortened RT is often associated with impulsive responses, which could explain deficits in social communications that are implicated in ASD. Individuals with ASD have the tendency to allocate limited amounts of cognitive resources to social stimuli and hasten to shift their attention to nonsocial stimuli. Consequently, individuals with high levels of autistic symptoms are less capable of properly processing the information in social communication and are not willing to interpret and feel other people’s actions and thoughts. The findings in the present study point to a general cognitive mechanism to account for multifaceted ASD symptoms.

Furthermore, our findings may account for the phenomenon of autistic savants [44]. That is, people who suffer from high ASD symptoms may exhibit an exceedingly high cognitive ability, which can be explained by the increased efficiency of information processing that was demonstrated by a faster response speed in our study. However, a shorter mu does not necessarily indicate better decision-making and problem-solving. Future studies should investigate the cognitive performance of individuals with high AQ scores in more complex tasks that require response speed and accuracy.

Unlike the ex-Gaussian mu, the parameter tau was not associated with the AQ or neuroticism score in the present study. Given that the parameter tau is thought to be linked to attentional control and top-down regulation [30,34], the null finding of ex-Gaussian tau may be due to the selection of the preclinical sample and the differences between diagnosed ASD and autistic traits. A key difference between ASD and autistic traits or preclinical autistic symptoms is that autistic traits or preclinical symptoms may occur among neurotypical people. As a result, individuals with preclinical autistic symptoms may not show altered cognitive processes, and the preclinical population does not receive a similar level of support to those with ASD, which in turn influences behavioral manifestations of the preclinical symptoms. Healthy young adults in the present study did not have deficits in the nervous system, and thus the ex-Gaussian tau of their RT performance was not impaired.

### Implications, Limitations, and Conclusion

The present study demonstrates a possible mechanism of the association between increased autistic traits and faster sensorimotor responses, suggesting that neuroticism should be included as an intervention target for ASD. Neuroticism is malleable. For example, children are still at the stage where their personalities are not stable and are sensitive to environmental factors. Therefore, optimizing the parenting style and school education are important for their traits’ development [45]. Moreover, among both children and adults, emotional regulation plays a central role in modifying neuroticism [46]. By increasing the usage of cognitive appraisal, individuals can strengthen inhibition control and improve symptoms of neuroticism [47]. Certain cognitive training and a program targeting physiological indicators of emotion regulation (e.g., heart rate variability, or HRV) can be used to reduce neuroticism and its negative behavioral outcomes [48]. In addition, mindfulness-based and other forms of cognitive therapy (MBCT) have been used to modify neuroticism [49,50].

The present findings also contribute to the identification of universal behavioral symptoms and traits in ASD across different cultural contexts. While cultural differences in social interactions that are associated with ASD are understudied [7], several studies have found differences in parent-reported and self-reported traits related to ASD between multinational groups [51,52]. Our findings suggest that the Big Five and objective behavioral measures (i.e., RT metrics) may reflect cognitive processes underlying various manifestations of autistic traits in different cultures.

The present findings need to be evaluated with consideration of several limitations. First, the sample in the present study consisted of healthy college students, and there were no clinical assessments for the sample. Although we focused on this population to address ASD prevention and the underreporting of ASD, preclinical autistic symptoms or traits may fundamentally be different from diagnosed ASD. Therefore, future studies should replicate the present findings in clinical samples. Second, there were no physiological or eye tracking data recorded during the choice RT task, so implicit attentional processes were not directly monitored. In research on psychopathology and emotion regulation, HRV and eye tracking have been widely used to study cognitive and affective processes that are associated with behavioral symptoms. Future studies should include those measures as well as direct neural indicators. Last, further research may utilize tasks involving social factors to examine information processing in social cognition among autistic individuals. As mentioned above, ASD and its symptoms are heterogeneous, so it is possible that the behavioral measures will show different patterns in a task that is designed to examine different aspects of the ASD symptoms.

In sum, we investigated a pathway through which high levels of autistic symptoms increase the response speed in a sensorimotor task among preclinical young adults. By analyzing the ex-Gaussian models of the RT data, different components of sensorimotor processes were derived. Neuroticism mediated the association between autistic symptoms and a faster speed of lower-level perceptual processing. The present findings contribute to the research on ASD and have practical implications for future intervention programs to improve daily functioning among individuals with ASD.

## Figures and Tables

**Table 1:** Demographic and descriptive statistics.

	Female (*n* = 69)	Male (*n* = 31)	Unadjusted *F* Statistics
Age (*M* years; *SD*)	20.29 (3.49)	20.39 (4.16)	0.02
Autistic Quotient (*M*; *SD*)	20.53 (7.54)	19.06 (6.49)	0.87
Neuroticism (*M*; *SD*)	8.06 (2.12)	6.66 (2.50)	8.36 **
Mean reaction time (*M* ms; *SD*)	397.27 (70.23)	378.32 (61.30)	1.63
Ex-Gaussian mu (*M* ms; *SD*)	322.34 (42.54)	322.69 (38.01)	0.01
Ex-Gaussian sigma (*M* ms; *SD*)	40.05 (14.35)	33.29 (13.77)	4.74 *
Ex-Gaussian tau (*M* ms; *SD*)	74.80 (59.40)	55.81 (30.49)	2.74

Note: Ex-Gaussian parameters were derived from ex-Gaussian modeling of reaction time data of the choice reaction time task. * *p* < 0.05; ** *p* < 0.01.

**Table 2:** Pearson correlations among variables.

	Autism Quotient	Neuroticism	Accuracy	mu	sigma	tau
Neuroticism	0.48 **					
Accuracy	−0.11	−0.05				
Ex-Gaussian mu	−0.23 *	−0.20	0.32 **			
Ex-Gaussian sigma	−0.01	0.10	0.05	0.62 **		
Ex-Gaussian tau	−0.11	−0.01	−0.26 *	0.14	0.27 **	
Mean reaction time	−0.06	−0.09	0.07	0.63 **	0.58 **	0.80 **

Note: Response accuracy and ex-Gaussian parameters mu, sigma, and tau were derived from the choice reaction time task, and they served as the indicators of behavioral performance. * *p* < 0.05; ** *p* < 0.01.

## Data Availability

The raw data supporting the conclusions of this article will be made available by the authors on request.
